# Systematic review for orthodontic and orthopedic treatments for anterior open bite in the mixed dentition

**DOI:** 10.1186/s40510-016-0142-0

**Published:** 2016-09-19

**Authors:** Lucia Pisani, Laura Bonaccorso, Rosamaria Fastuca, Raffaele Spena, Luca Lombardo, Alberto Caprioglio

**Affiliations:** 1 Division of Orthodontics, Department of Surgical and Morphological Sciences, University of Insubria, Varese, Italy; 2 Postgraduate School of Orthodontics, University of Ferrara, Ferrara, Italy

**Keywords:** Early treatment, Open bite, Systematic review, Quality analysis

## Abstract

**Background:**

The treatment options for the early treatment of anterior open bite are still controversial. The aim of this study was to evaluate the actual available evidence on treatments of anterior open bite in the mixed dentition in order to assess the effectiveness of the early treatment in reducing open bite, the most efficacious treatment strategy and the stability of the results.

**Materials and methods:**

A literature survey was done on November 15, 2015, by means of appropriate Medical Subject Headings (MeSH) using the following databases: PubMed, EMBASE, Cochrane Library, LILACS, VHL, and WEB OF SCIENCE.

Randomized clinical trials and studies with a control group (treated or untreated) were then selected by two authors. Trials including patients with syndromes or in the permanent dentition and studies concerning treatment with extractions, full-fixed appliances, or surgery were not considered.

Full articles were retrieved for abstracts or titles that met the initial inclusion criteria or lacked sufficient detail for immediate exclusion.

**Results:**

Two thousand five hundred sixty-nine studies about open bite were available; the search strategy selected 240 of them.

Twenty-four articles have been judged suitably for the final review, and their relevant data were analyzed.

**Discussion:**

Although this review confirms the effectiveness of early treatment of open bite, particularly when no-compliance strategies are employed, meta-analysis was unfeasible due to lack of standardization, important methodological limitations, and shortcomings of the studies.

**Conclusions:**

A more robust approach to trial design in terms of methodology and error analysis is needed. Besides, more studies with longer periods of follow-up are required.

## Review

### Background

Anterior open bite is a malocclusion characterized by a deficiency in the normal vertical overlap between antagonist incisal edges when the posterior teeth are in occlusion [1].

Dental and dentoalveolar open bite is the result of a mechanical blockage of the vertical development of the incisors and the alveolar component while skeletal relationships are normal; skeletal open bite is determined by a vertical skeletal discrepancy [2]. However, in most cases, the distinction is not clear since malocclusion presents both dental and skeletal components [3].

Skeletal open bite is characterized by increased lower anterior facial height and gonial angle, short mandibular ramus, and increased posterior dentoalveolar height. Concomitant transverse discrepancies may also be present [4]. Additional features are lip incompetence, profile convexity, marked incisors labial inclination and crowding [5, 6]. For these reasons, anterior open bite is a major cause of masticatory and phonatory function impairment and also causes considerable esthetic issues to the affected patients [7].

Etiology involves the interaction of environmental factors such as prolonged sucking habits, mouth breathing, tongue or lip thrusting, and eruption disturbances with a genetically determined vertical facial grow pattern [2, 6, 8–13].

Several authors emphasized that a skeletal open bite should be treated in the mixed dentition in order to take advantage of the active growth producing faster and more stable results and to reduce the burden of treatment in the permanent dentition [14, 15]. Various approaches have been proposed on this purpose.

Vertical chin cup [16], bite blocks [17–24], chewing exercises [25], and extractions and mesialization of posterior teeth [26] have been advocated to achieve relative and true intrusion of molars. Palatal cribs and spurs are used to prevent persisting sucking habits or tongue thrust in order to promote a normal anterior segment development [27–35]. Functional therapy would be useful in correcting the faulty postural activity of the orofacial musculature and the associated skeletal deformity [36–41].

However, treatment of skeletal anterior open bite is still one of the most difficult challenges for the orthodontist. Effectiveness and long-term stability of available treatment modalities are critical issues because of the lack of a strong scientific evidence [42, 43].

The objective of this work was to perform a systematic review of the literature in order to evaluate the actual available evidence on treatments of anterior open bite in the mixed dentition and to assess the effectiveness of the early treatment in reducing open bite and divergency, the most efficacious treatment strategy and the stability of the results.

### Materials and methods

This systematic review was written according to the PRISMA guidelines [44].

The search strategy was based on the National Health Service Center for Reviews and Dissemination guidelines [45].

A first survey of all articles published up to November 2015 about anterior open bite was performed by using the following databases: PubMed, EMBASE, Cochrane Library, LILACS, VHL, and WEB OF SCIENCE.

The search strategy for PubMed was then improved according to Cochrane Collaboration guidelines using the Medical Subject Headings (MeSH) terms “early treatment” and “dentition, mixed,” crossed with combinations of the MeSH term “open bite”.

The key words used to identify the corresponding studies in the other databases were: “open bite” and “mixed dentition”.

References from original papers and reviews were checked.

Randomized controlled trials (RCTs) and prospective or retrospective studies with a control group (treated or untreated) reporting data on the effects of the treatment in the mixed dentition were included.

Descriptive studies, case reports, case series, debate articles, and studies concerning treatment in the permanent dentition, with extractions, with full-fixed appliances, or surgically assisted were excluded. Studies including patients with cleft lip or palate or both or other syndrome associated with craniofacial anomalies were not considered.

Duplicate reports were excluded.

Two authors (L.P. and L.B.) screened the titles and abstracts and independently assessed the eligibility of all the reports. Full articles were retrieved for abstracts or titles that met the initial inclusion criteria or lacked sufficient details for immediate exclusion.

The articles that were judged suitably for the final review analysis were read, and their relevant data were retrieved for pooling.

Data were collected on study design, treatment modalities, characteristics of the sample, methods of measurements, success rate, decrease of open bite and divergency, treatment duration, side effects and costs, and stability.

A quality evaluation modified by the protocol described by Antczak [46] and Jadad [47] was performed for each article. This considered sample size, selection description, withdrawals, validity of the methods, method error analysis, blinding in measurements, and adequate statistics. The quality was categorized as low, medium, and high.

### Results

**Fig. 1:**
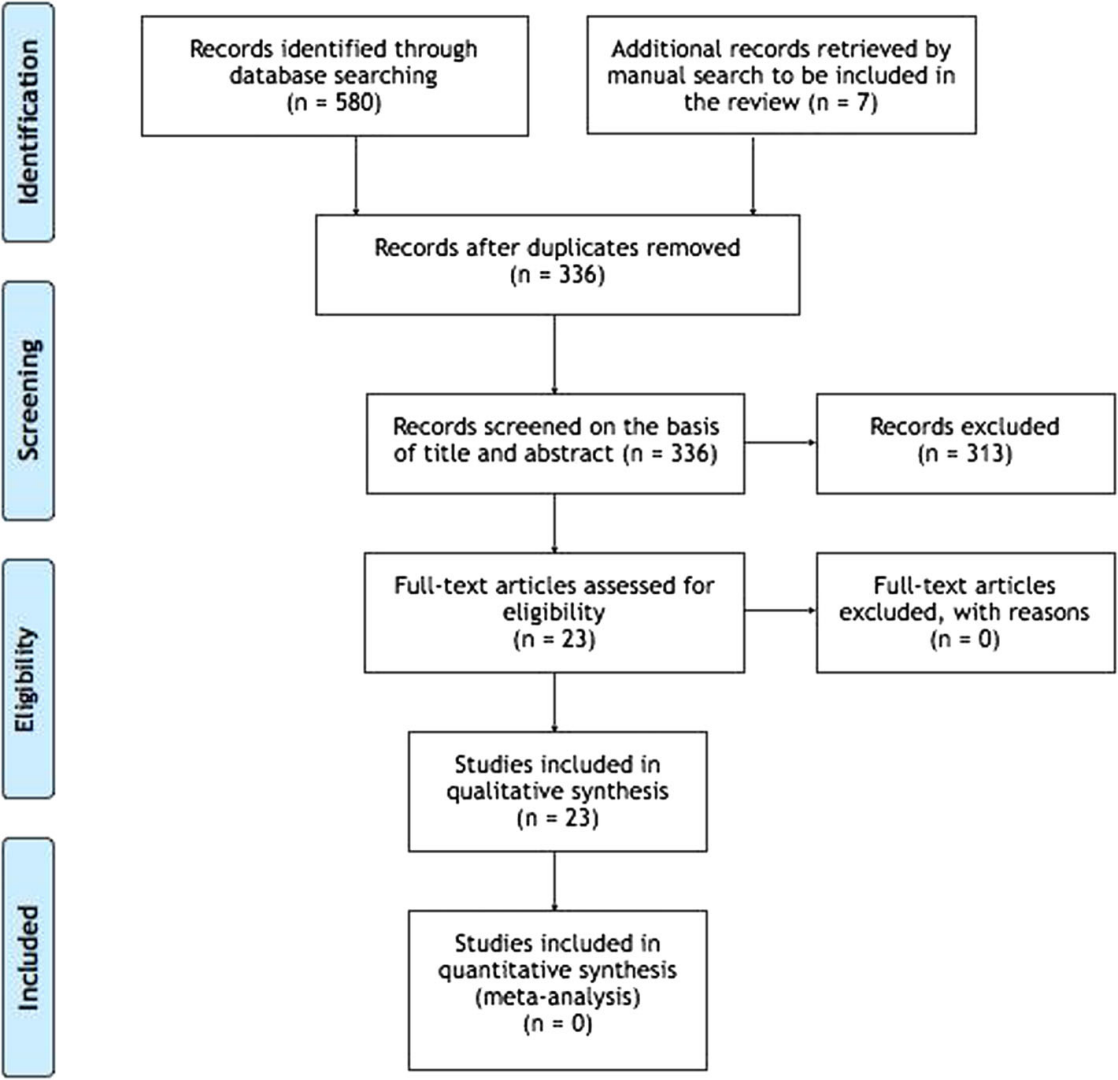
Flow chart of the selection of the studies

As shown in the flow chart (Fig. 1), 2569 articles about anterior open bite were available in the literature.

The combination of MeSh terms resulted in 240 articles.

According to the inclusionary/exclusionary criteria, 25 articles, published between 1983 and 2015, were selected.

One was excluded because two groups were treated with a multibracket appliance.

Twenty four articles were then considered for the final review analysis.

#### Trial design and treatment modalities

Data about trial design and treatment modalities are shown in Table 1.

**Table 1:** Results: trial design, treatment modalities, characteristics of the samples

Selected references Year of publication Study design	Treatment modalities	Sample size	Age (years)	Sex
Albogha H et al. [24]	RMI vs. MBB	15 (RMI)	8.1–13.5 (RMI)	4M, 11F (RMI)
2015		15 (MBB)	8.5–13.5 (MBB)	6M, 9F (MBB)
P, L, CT				
Mucedero M et al. [35]	Q-H/C vs. UCG	28 (Q-H/C)	8.2 ± 1.3 (Q-H/C)	11M, 7F (Q-H/C)
2013		20 (UCG)	8.1 ± 0.4 (UCG)	10M, 10F (UCG)
R, L, CCT				
Torres FC et al. [33]	RPC + VCC vs. FPC + VCC	30 (RPC + VCC)	8.33 ± 0.73 (RPC + VCC)	8M, 22F (RPC + VCC)
2012		30 (FPC + VCC)	8.54 ± 0.88 (FPC + VCC)	11M, 19F (FPC + VCC)
P, L, CT				
Cassis MA et al. [34]	BS + VCC vs. UCG	30 (BS + VCC)	8.14 ± 0.73 (BS + VCC)	9M, 21F (BS + VCC)
2012		30 (UCG)	8.36 ± 1.05 (UCG)	30.5M, 25F (UCG)
P, L, CCT				
Doshi UH et al. [23]	SLBB vs. MBB	10 (SLBB)	8–13(SLBB)	5M, 5F (SLBB)
2010		10 (MBB)	8–13 (MBB)	3M, 7F (MBB)
P, L, RCT		10 (UCG)	8–13 (UCG)	
Giuntini V et al. [32]	Q-H/C vs. RPC	20 (Q-H/C)	8.4 ± 1.4 (Q-H/C)	5M, 15F (Q-H/C)
2008		20 (RPC)	8.4 ± 1 (RPC)	9M, 11F (RPC)
R, L, CT				
Cinsar A et al. [22]	RMI vs. UCG	10 (RMI)	M 11 ± 0.4;	3M, 7F (RMI)
(subgroups)		10 (UCG)	F 10.3 ± 0.2	3M, 7F (UCG)
2007			(RMI)	
R, L, CCT				
			M 11 ± 1;	
			F 10.8 ± 0.9 (UCG)	
Defraia E et al. [41]	OBB vs. uCG	20 (OBB)	8.2 ± 0.8 (OBB)	11M, 9F (OBB)
2007		23 (UCG)	10.8 ± 1.5 (UCG)	23 (UCG)
R, L, CCT				
Cozza P et al. [30]	Q-H/C vs. UCG	21 (Q-H/C)	8.4 ± 1.5 (Q-H/C)	6M, 15F (Q-H/C)
2007		21 (UCG)	8.6 ± 11M (UCG)	10M, 11F (UCG)
R, L, CCT				
Cozza P et al. [31]	Q-H/C vs. OBB	21 (Q-H/C)	8.4 ± 1.4 (Q-H/C)	6M, 15F (Q-H/C)
2007		20 (OBB)		
R, L, CT				
Pedrin F et al. [27]	RPC + VCC vs. UCG	30(RPC + VCC)	8.61 (RPC + VCC)	10M, 20F (RPC + VCC)
2006		30 (UCG)	8.33 (UCG)	7M, 23F (UCG)
P, L, CCT				
Torres F et al. [28]	RPC + VCC vs. UCG	30(RPC + VCC)	8.33 (RPC + VCC)	8M, 22F (RPC + VCC)
2006		30 (UCG)	8.61 (UCG)	7M, 23F (UCG)
P, L, RCT				
Cozza P et al. [29]	Q-H/C vs. UCG	23 (Q-H/C)	8.4 ± 1.4 (Q-H/C)	7M, 16F (Q-H/C)
2006		23 (UCG)	9.1 ± 1.6 (UCG)	10M, 13F (UCG)
R, L, CCT				
Iscan HN et al. [16]	VCC vs. UCG	18 (VCC)	8.08–11.11 (VCC)	6M, 12F (VCC)
2002		17 (UCG)	8.40–12.26 (UCG)	6M, 11F (UCG)
P, L, CCT				
Bazzucchi A et al. [21]	MBB vs. UCG	29 (MBB)	11.08 ± 3.08 (MBB)	6M, 23F (MBB)
1999		29 (UCG)	11 ± 3.08 (UCG)	6M, 23F (UCG)
R, L, CCT				
Iscan HN and Sarisoy L [20]	PBB5 vs. PBB10 vs. UCG	13 (PBB5)	8.9–13.5 (PBB5)	4M, 9F (PBB5)
1997		12 (PBB10)	8.7–14.5 (PBB10)	3M, 9F (PBB10)
P, L, CCT		14 (UCG)	8.9–13.6 (UCG)	3M, 11F (UCG)
Erbay E et al. [40]	FR + LSE vs. UCG	20 (FR + LSE)	8.7 ± 0.5 (OBB)	7M, 13F (FR + LSE)
1995		20 (UCG)	8.9 ± 1.2 (UCG)	7M, 13F (UCG)
P, L, RCT				
Iscan HN et al. [19]	SLBB vs. PBB + VCC	11 (SLBB)	8.62–13.54 (SLBB)	Not declared
1992		12 (PBB + VCC)	7.39–11.67 (PBB + VCC)	
R, L, CT				
Weinbach JR and Smith RJ. [39]	OBB vs. UCG	26 (OBB)	7.08–12.88	27M, 12F
1992		13 (OBB + HPH)		
R, L, CCT		Published normal growth standards		
Kuster R and Ingervall B [18]	SLBB vs. MBB	22 (SLBB)	7.4–11.56(SLBB)	11M, 11F (SLBB)
1992		11 (MBB)	9.72–14.4 (MBB)	4M, 7F (MBB)
R, L, CT				
Ngan P et al. [38]	A-HPH vs. UCG	8 (A-HPH)	10.24(A-HPH)	2M, 6F
1992		8 (UCG)	10.24 (UCG)	
R, L, CCT				
Haydar B and Enacar A [37]	FR + LSE vs. UCG	11 (FR)	8.8 ± 1.17 (OBB)	Not available
1992		10 (UCG)	8.3 ± 1.06 (UCG)	
P, L, CCT				
Kiliaridis S et al. [17]	MBB vs. PBB	10 (MBB)	8.9–16.1	3M, 7F (MBB)
1990		10 (PBB)		3M, 7F (PBB)
P, L, CT				
Frankel R [36]	FR + LSE vs. UCG	30 (FR)	7 (FR)	Not declared
1983		11 (UCG)	8 (UCG)	
R, L, CCT				

*Legends*: Study design: *P* prospective, *L* longitudinal, *CT* clinical trial, i.e., comparison of at least two treatment modalities without any untreated or normal group involved, *R* retrospective, *CCT* controlled clinical trial, *RCT* randomized controlled trial. Treatment modalities: *RMI* rapid molar intruder, *MBB* magnetic bite block, *Q-H/C* quad-helix/crib, *UCG* untreated control group, *RPC* removable palatal crib, *VCC* vertical chin cup, *FPC* fixed palatal crib, *BS* bonded spurs, *SLBB* spring-loaded bite block, *OBB* open bite bionator, *PBB5* posterior bite blocks 5 mm, *PBB10* posterior bite blocks 10 mm, *FR* Fränkel appliance, *LSE* lip seal exercises, *A-HPH* Teuscher appliance. Sex: *M* male, *F* female

Three randomized controlled trials were found [23, 28, 40].

The effects of Quad Helix with crib (Q-H/C) were examined by three studies [29, 30, 35]. Other trials compared them versus those of removable palatal crib (RPC) [32] and open bite bionator (OBB) [31].

OBB was tested by two further authors alone [16, 41] in combination with a high-pull headgear (HPH) [39]. Two studies described the effects of RPC associated with a vertical chin cup (RPC + VCC) [27, 28] and one compared it versus fixed palatal crib and VCC (FPC + VCC) [33].

One trial assessed the effects of bonded spurs in combination with VCC (BS + VCC) [34], one those of Teucher appliance (A-HPH) [38], and one those of VCC alone [16].

The results of Fränkel appliance (FR) was described by three studies [19, 36, 40].

Posterior bite blocks at 5 or 10 mm in height (PBB5, PBB10) [20] and magnetic bite blocks (MBB) [21] were tested. The effects of MBB were compared versus spring-loaded bite blocks (SLBB) [18, 23], PBB [37], and rapid molar intruder (RMI) [24].

This latter was tested versus a control group in one trial [22] where a further group enrolled older patients treated with RMI and a multibracket appliance. Since the application of a multibracket appliance was not suitable with our inclusion criteria, only data relative to RMI group and control group were considered.

Finally, Işcan et al. compared the association PBB + VCC versus SLBB [19].

#### Characteristics of the participants

Five authors [27, 28, 33, 34, 40] included only the subjects with anterior open bite greater than 1 mm.

Eighteen studies [16–20, 22–24, 29–32, 35–38, 40, 41] had skeletal anterior open bite in their inclusion criteria. For the remaining studies [21, 27, 28, 33, 34, 39], the inclusion criterion was anterior open bite independently of the type.

Bad habits were an exclusion criterion in five studies [17, 20, 22, 28, 37] while four trials [29, 30, 32, 35] included only patient with thumb-sucking habit and related constricted maxillary arch before treatment. The remaining studies did not evaluate the presence of bad habits.

Three articles [22, 28, 34] excluded subjects with maxillary constriction.

Full eruption of the permanent first molars and incisors was an inclusion criterion for the seven studies [19, 20, 29, 30, 33–35] to prevent the “pseudo-open bite” due to under-erupted permanent incisors.

Other inclusion criteria considered by few authors were no teeth absence due to ageneses or extractions [19, 33–35, 40], no previous orthodontic treatment [33], no crowding [27, 28, 33, 34], no need for adenoidectomy or tonsillectomy [16, 19, 20, 28], excessive overjet [38], anterior open bite unchanged or increased in the last 6 months [17], large interlabial distance, and postural weakness of the orofacial muscles [36]. These aspects were not examined by the other authors.

Most of the trials selected patients with anterior open bite regardless their skeletal and molar class.

Details about sample size, age, and sex of the participants were resumed in Table 1.

Sexual dimorphism was evaluated and not found in three studies [27, 28, 34] while it was found for few parameters in one study [40].

#### Success rate

The success rate was 100 % in four studies [17, 19, 22, 38], 80–90 % in eight studies [16, 27–30, 34, 35, 41], and 67 % in one study [39] with untreated control group (Table 2).

**Table 2:** Results: success rate, treatment duration, reduction of open bite and divergency, side effects and stability

Selected references	Success rate	Treatment duration/observation time	Time of daily appliance wear (h)	Reduction of open bite and divergency	Methods of measurement	Side effects	Follow-up/stability
Albogha H et al. [24]	33 % (RMI)	4 months	24 h	Yes open bite	Cephalometry	Both hindered oral hygiene.	No/no stability information
	27 % (MBB)			Yes divergency			
Mucedero M et al. [35]	86 %	18 months/no retention information	24 h	Yes open bite	Cephalometry	No	At least 5 years/no relapse (data not suitable with inclusion criteria)
				Yes divergency			
Torres FC et al. [33]	70 % (FPC + VCC)	12 months/no	24 h (RPC, FPC)	Yes open bite	Cephalometry	No	No/no stability information
	50 % (RPC + VCC)	Retention information	14-16 h (VCC)	No divergency			
Cassis MA et al. [34]	86.7 %	12 months/no retention information	24 h (BS)	Yes open bite	Cephalometry	No	No/no stability information
			14-16 h (VCC)	Yes divergency			
Doshi UH et al. [23]	Not declared	Until an edge-to-edge bite was achieved (max 8 months)/retention with passive BB for 10 months	not declared	Yes open bite	Clinical evaluation, cephalometry, electromyography	Broken spring replaced in 7 pz (SLBB)	10 months/insignificant dentoalveolar relapse
				Yes divergency			
Giuntini V et al. [32]	90 % (Q-H/C)	18 months/no retention information	24 h (Q-H/C)	Yes open bite	Cephalometry	No	No/no stability information
	60 % (RPC)		16 h (RPC)	Yes divergency			
Cinsar A et al. [22] (subgroups)	100 %	9–11 months/no retention information	24 h	Yes open bite	Cephalometry	No	No/no stability information
				Yes divergency			
Defraia E et al. [41]	85 %	18 months/about 12 months of retention with OBB	24 h	Yes open bite	Cephalometry	No	No/no stability information
				Yes divergency			
Cozza P et al. [30]	85 %	18 months/no retention (16 patients); removable appliance for retention for 1 year (5 patients)	24 h	Yes open bite	Cephalometry	No	2 years/relapse in 15 % of subjects
				Yes divergency			
Cozza P et al. [31]	Not declared	Active treatment	24 h (Q-H/C)	Yes open bite	Cephalometry	No	1 year/no relapse (QH/C)
		18 months/no retention (Q-H/C)	24 h (OBB)	Yes divergency (QH/C more than OBB)			
							No stability information (OBB)
		18 months/no retention, with the exception of a few patients who continued to use the OBB at night (OBB)					
		Observation time					
		2.6 years ± 9 months (Q-H/C)					
		2.5 years ± 1.2 years (UCG)					
Pedrin F et al. [27]	80 %	12 months/no retention information	14–16 h	Yes open bite	Cephalometry	No	No/no stability information
				No divergency			
Torres F et al. [28]	80 %	12 months/no retention information	14–16 h	Yes open bite	Cephalometry	No	No/no stability information
				No divergency			
Cozza P et al. [29]	90 %	18 months/no retention information	24 h	Yes open bite	Cephalometry	No	No/No stability information
				Yes divergency			
Işcan HN et al. [16]	88 %	6–12 months until overbite was obtained (mean 9 months) no retention	16 h	Yes open bite	Cephalometry	No	No/no stability information
				Yes divergency			
Bazzucchi A et al. [21]	Not declared	8 months (MBB)	Not declared	Yes openbite	Cephalometry	No	Not suitable with inclusion criteria
		9 months (uCG)		Yes divergency			
		No retention information		(Not statistically but clinically significant changes)			
Işcan HN and Sarisoy L [20]	80 % (PBB5)	4–10 months, until an overbite of 1–1.5 mm was achieved (PBB5)	18 h	Yes open bite	Cephalometry	No	No/no stability information
	66 % (PBB10)			Yes divergency			
		4–13 months, until an overbite of 1–1.5 mm was achieved (PBB10)					
		7–9 months (UCG)					
		No retention information					
Erbay E et al. [40]	Not declared	24 months (FR)	18 h	Yes open bite	Cephalometry	No	No/no stability information
		24 months (UCG)		Yes divergency			
		No retention information					
Işcan HN et al. [19]	100 %	1–10 months until an overbite of 1–1.5 mm was achieved (SLBB)	16 h	Yes open bite	Cephalometry	No	No/no stability information
				Yes divergency			
		3–9 months until an overbite of 1–1.5 mm was achieved (PBB) then worn only at night for retention					
Weinbach JR and Smith RJ [39]	67 % had a reduction of open bite	Mean 20 months	Not declared	Yes open bite	Cephalometry	No	No/no stability information
		No retention		Yes divergency			
Kuster R and Ingervall B [18]	Not declared	SLBB 1 year	At night (SLBB)	Yes open bite	Bite force, cephalometry, electromyography	Broken spring replaced in 12 pz (SLBB)	6 months/tendency to relapse (MBB)
		MBB 3 months	24 h (MBB)	Yes divergency			
		2 MBB patients: no retention					
		1 MBB: activator as retention for 1 year					
							No stability information (SLBB)
		3 MBB patients: upper removable plate with posterior platforms 6–8 months					
		3 MBB patients 1 year multibanded appliance					
Ngan P et al. [38]	100 %	Mean 14 months until overcorrection of dental and skeletal relationship	2 h (first 3 days) than increased until 24 h (A)	Yes open bite	Cephalometry	No	No/no stability information
				Yes divergency	Study casts		
		No retention	12–14 h (HPH)				
Haydar B and Enacar A [37]	Not declared	FR 1235 years	Not available	Yes open bite	Cephalometry	No	No/no stability information
		UCG 1024 years		No divergency			
Kiliaridis S et al. [17]	100 %	6 months	18 h	Yes open bite	Cephalometry, study casts, intra-oral photographs, monthly analysis of the stomatognatic system	Lateral crossbite (MBB)	No/no stability information
		No retention		Yes divergency			
						Effect declined with time (PBB)	
Fränkel R et al. [36]	Not declared	No treatment and retention durations information	Not declared	Yes open bite	Cephalometry	No	At least 4 years out of retention/Stability if lips
		Observation time		Yes divergency			Sealed without muscular straint.
		8 years					Relapse rate not declared

*Legends*: *RMI* rapid molar intruder, *MBB* magnetic bite block, *Q-H/C* quad-helix/crib, *UCG* untreated control group, *RPC* removable palatal crib, *VCC* vertical chin cup, *FPC* fixed palatal crib, *BS* bonded spurs, *SLBB* spring-loaded bite block, *OBB* open bite bionator, *PBB5* posterior bite blocks 5 mm, *PBB10* posterior bite blocks 10 mm, *FR* Fränkel appliance, *LSE* lip seal exercises, *A-HPH* Teuscher appliance

Seven studies omitted the success rate [18, 21, 23, 31, 36, 37, 40].

A 20–30 % difference in the success rate was found in two studies which compared two different treatment modalities [32, 33]. Lower differences were found in two studies [20, 24].

#### Treatment duration and open-bite reduction

The treatment duration varied significantly among the different study protocols (Table 2), and it was not declared in one study [36].

All the trials observed a reduction of the open bite in the treatment group. Bazzucchi [21] reported no statistically significant changes between treated subjects and controls even if dental and skeletal changes were found to be clinically relevant.

In 20 studies, the treatment had also skeletal effects [16–24, 29–32, 34–36, 38–41].

The amount of open-bite reduction varied from 3.1 to 5.1 mm for RPC, alone [32] or in association with VCC [27, 33], and from 4.1 to 5.44 mm for fixed cribs as FPC [33], Q-H/C [29–31, 35], BS [34] with [33, 34] or without [30–32, 35] VCC.

Işcan found that the VCC alone produced 3.92 mm of overbite correction [16].

The mean correction of the overbite achieved with bite blocks varied from 2.25 to 4.58 mm for PBB [19, 20, 37], from 1.3 to 3.59 mm for SLBB [18, 19, 23], from 2.00 to 4.9 mm for MBB [17, 21, 23, 24, 35], and from 3.1 to 4.55 for RMI [22, 24].

OBB showed a mean correction of overbite varying from 1.3 to 2.7 mm.

FR was used in three studies [36, 37, 40] which reported a reduction of the overbite varying from 2.63 to 5 mm due to the therapy.

The only trial about A-HPH [38] did not declare the amount of correction of the open bite (Table 3).

**Table 3:** Quality analysis

Article	Previous estimate of sample size	Selection description	Withdrawals	Valid method	Method error analysis	Blinding in measurements	Adequate statistics provided	Judge quality standard
Albogha H et al. [24]	Not	Adequate	Not known	Partly	Yes	Not	Yes	Low
Mucedero M et al. [35]	No/not known	Adequate	Not known	Yes	Yes	Not	Yes	Medium
Torres FC et al. [33]	No/not known	Adequate	Not known	Yes	Yes	Not	Yes	Medium
Cassis MA et al. [34]	Yes	Adequate	Not known	Yes	Yes	Not	Yes	Medium
Doshi UH et al. [23]	Not	Adequate	Not known	Partly	Yes	Not	No	Low
Giuntini V et al. [32]	Not	Adequate	Not known	Yes	Yes	Not	Yes	Medium
Cinsar A et al. [22] subgroup.	Not	Adequate	Not known	Yes	Yes	Not	Yes	Medium
Defraia E et al. [41]	Not	Adequate	Not known	Yes	Yes	Not	Yes	Medium
Cozza P et al. [30]	Not	Adequate	Not known	Yes	Yes	Not	Yes	Medium
Cozza P et al. [31]	Not	Adequate	Not known	Yes	Yes	Not	Yes	Medium
Pedrin F et al [27]	Not	Adequate	None	Partly	Yes	Not	Not	Low
Torres FC et al. [28]	Yes	Adequate	Not known	Partly	Yes	Not	Not	Medium
Cozza P et al. [29]	Not	Adequate	Not known	Partly	Yes	Not	Yes	Low
Işcan HN et al. [19]	Not	Adequate	Not known	Yes	Not	Not	Not	Low
Bazzucchi A et al. [21]	Not	Adequate	Not known	Not	Yes	Not	Not	Low
Işcan HN and Sarisoy L [20]	Not	Adequate	Not known	Partly	Yes	Not	Yes	Medium
Erbay E et al. [40]	Not	Adequate	Not known	Partly	Not	Not	Yes	Medium
Işcan HN et al. [19]	Not	Adequate	One	Yes	Yes	Not	Inadequate	Low
Weinbach JR and Smith RJ [39]	Not	Adequate	Not known	Not	Not	Not	Inadequate	Low
Kuster R and Ingervall B [18]	Not	Adequate	One	Yes	Yes	Not	Yes	Medium
Ngan P et al. [38]	Not	Adequate	Not known	Yes	Yes	Not	Inadequate	Low
Haydar B and Enacar A [37]	Not	Adequate	Not known	Partly	Not	Not	Yes	Medium
Kiliaridis S et al. [17]	Not	Adequate	Four	Partly	Yes	Yes	Absent	Low
Fränkel R [36]	Not	Adequate	Not known	Partly	Not	Not	Yes	Medium

#### Side effects and costs

Regarding side effects, one study declared that RMI and MBB hindered oral hygiene [24].

Although no spurs were lost during the treatment period, Cassis reported that the possibility to fall and being aspired into the lungs or swallowed should be considered in the appliance selection [34].

In two trials [18, 23], more than half SLBBs were broken during the treatment.

One study reported that unilateral crossbite occurred in half of the patients who wore MBB extensively [17].

The disadvantage of the PBB is that treatment effects declined over time, possibly because of a decrease in the force applied to the antagonist teeth by the mandibular elevator muscles [19].

Işcan found that increasing the height of PBB resulted in an increase in the gonial angle probably because of a muscular response to the artificially increased vertical dimension and suggested that this angle should be examined in the long term [20].

One study reported that FR appliance caused an unexpected backward rotation of the mandible in the treated group [37].

No studies performed a cost analysis.

#### Stability

Eighteen studies did not analyze treatment stability [16, 17, 19–22, 24, 27–29, 32–34, 37–41]. Three studies found insignificant or absent relapse [23, 31, 35].

Mucedero [35] reported stability after at least 5 years from the end of the treatment. These data are not suitable with our inclusion criteria since a fixed appliance was used during the follow-up period.

Cozza [30] evaluated the treated group of a previous study [29] 2 years after the active treatment finding relapse in 15 % of the subjects.

Kuster and Ingervall [18] did not provide stability information about the treatment with SLBB, while they reported a tendency to relapse for MBB group after 1 year. Fränkel [36] reported that when open bite was associated with an hyperdivergent skeletal pattern, relapse occurred in all treated cases unless a competent anterior oral seal had been achieved.

However, these last two studies did not declare the relapse rate (Table 3).

#### Quality analysis

Since several items required in quality reviews [46, 47] were not applicable to this study, the quality of the articles was judged as low, medium, or high as proposed by Petrén et al [48].

Most studies presented shortcomings, problems of selection, and misuration bias.

Research quality was low in ten studies [16, 17, 19, 21, 23, 24, 27, 29, 38, 39] and medium in 14 (Table 3).

Due to the insufficient number of RCTs, the lack of standardization of diagnostic criteria, inclusion criteria, validity measures to evaluate outcomes, and methodological limitations, a meta-analysis could not be performed.

### Discussion

Recently, Feres et al. [42] performed a systematic review on the effectiveness of the open-bite treatment in growing children and adolescents concluding that consistent results were not found. A further review by Lentini-Oliveira published in 2014 including only RCTs assessed that there were no clear evidence on which to make a clinical decision of the type of intervention to use [43].

Besides, the present study aims to focus wholly on the open-bite treatment of subjects in the mixed dentition. The authors included also not RCTs, since in their opinion, their analysis could lead to significant outcomes.

Several treatment modalities were studied and their effects are summarized in Table 4.

**Table 4:** Summary of effects

Treatment modalities		Summary of effects	Reduction of open bite	Reduction of divergency
Rapid molar intruder (RMI)		Molar intrusion	Yes	Yes
		Mandibular autorotation		
Bite blocks	Magnetic bite block (MBB)	Incisors extrusion, molar intrusion	Yes	Yes
		Control of mandibular skeletal height		
		Mandibular autorotation		
		Lateral crossbite		
		More effective than spring loaded bite blocks		
		Faster and more effective than acrylic bite blocks		
	Spring-loaded bite block (SLBB)	Incisors extrusion, maxillary molar intrusion	Yes	Yes
		Control of posterior dentoalveolar height		
		Mandibular autorotation		
		Tendency to break		
		Greater ramal inclination and molar intrusion than acrylic bite blocks		
	Posterior bite blocks 5 mm (PBB5); posterior bite blocks 10 mm (PBB10)	Incisive extrusion and lingual tipping, molar intrusion	Yes	Yes
		Control of posterior dentoalveolar height		
		Mandibular autorotation		
		PBB5 and PBB10 are both effective		
		PBB10 produce greater mandibular sagittal growth and autorotation, increase of gonial angle		
Quad-helix/crib (Q-H/C)		Stop sucking habits	Yes	Yes
		Incisors extrusion and lingual tipping		
		More efficient than removable cribs since it does not need for compliance		
		Downward rotation of palatal plane and improvement of intermaxillary vertical relationships		
Cribs or spurs	Fixed palatal crib (FPC)	More efficient than removable cribs since it does not need for compliance	Yes	Data in disagreement
	Removable palatal crib (RPC)	Just anterior dento-alveolar effects (extrusion and verticalization of maxillary and mandibular incisors)	Yes	Data in disagreement
		Molar eruption not controlled		
		Skeletal effects depend on patient’s compliance		
	Spurs (BS)	Dentoalveolar effects	Yes	Yes
Vertical chin cup (VCC)		Reduction of open bite	Yes	Data in disagreement
		Molar eruption not controlled		
		Skeletal effects depend on patient’s compliance		
Functional appliances	Open bite bionator (OBB)	Useful for class II open bite malocclusions	Yes	Yes
		Control of maxillary molars extrusion		
		Improvement of intermaxillary vertical relationships		
	Fränkel appliance + lip seal exercises (FR + LSE)	Dentoalveolar effects, upper incisors lingual tipping	Yes	Data in disagreement
		Stability if lips sealed without muscular straint		
		Data about skeletal effects are in disagreement		
	Teuscher appliance (A-HPH)	Effective for class II open bite malocclusions	Yes	Yes
		Lingual tipping of maxillary incisors		
		Reduction of forward growth of the maxilla		
		Control of maxillary molars extrusion and mesialization		
		Increase of mandibular alveolar height		

The analysis of the results suggests that early treatment was able to intercept and reduce dentoskeletal open bite, in particular when it was caused by an altered function.

VCC alone [16] or associated with other devices [27, 28, 33, 34] produced an increase of the overbite. Although the same protocol of use and similar samples in the studies, some authors [27, 28] showed that VCC did not yield favorable skeletal effects, and others [16, 34] reported vertical control and decreased gonial angle probably because of greater compliance.

For the same reason, fixed palatal cribs (FPC; Q-H/C) showed a greater amount of overbite improvement compared to removable appliances [31–33].

On the other hand, RPC + VCC produced a greater improvement in overjet as a result of activations and adjustments.

The therapy with RPC depends on the patient compliance, but, in many cases, it provides a greater comfort than the FPC because it can be worn gradually and can be removed for meals and oral hygiene, which would be favorable from the psychological point of view.

Cribs were found to produce a clinically significant improvement in the maxillomandibular vertical relationships by some authors [29–32, 34, 35], while others [27, 28, 33] reported only dental effects.

OBB showed an improvement of intermaxillary vertical relationships [31, 39, 41] even if less than Q-H/C [31] and proved to be useful for class II open-bite patients since it reduced facial convexity, ANB angle, and overjet and restricted maxillary molar extrusion, achieving vertical control. The association with a HPH had no significant effect compared with the bionator alone [39].

Bite blocks were found to improve the divergency except for the SLBB tested by Kuster and Ingervall which had just dental effects [18].

For the PBB, the mean change in overbite was less than 3 mm when used alone [17, 20], 4.6 mm when used with VCC [19].

Işcan [20] found that higher PBB were not more effective in improving overbite compared to shorter PBB, but they had greater favorable effects on the sagittal growth and mandibular anterior rotation.

Işcan [19] also demonstrated that SLBB produced greater ramal inclination and molar intrusion than PBB + VCC even if the amount of correction of the open bite was smaller. Both therapies led also to upward and forward mandibular autorotation and decrease the anterior facial height.

Due to greater dentoalveolar and skeletal effects, MBB proved to be more effective than SLBB [18, 23], PBB [17], and RMI [24].

The MBB elicited significantly greater decreases in the SNA and ANB angles, maxillary incisor angle, and overjet compared with RMI. This can be attributed to the fact that the deformation of the elastic modules of RMI reduced the applied force over time, while it was consistent for magnets. Patients with MBB had then to apply more muscular tension to achieve a lip seal with greater effects attributable to labial pressure. This suggests that MBB might be preferred for open-bite class II with protrusion of the maxillary incisors [24].

Two studies which tested FR + LSE reported an upward and forward mandibular rotation in the treated group, whereas backward rotation continued in the control sample [36, 40]. On the contrary, Haydar and Enacar [37] denied favorable skeletal effects and assessed an unexpected slight mandibular posterior rotation.

A-HPH was tested in patients with class II skeletal open bite, and it proved to correct open bite and molar relationships due to both favorable dentoalveolar and skeletal effects [38].

#### Quality of the studies

Randomization increases the reliability of a study and allows final differences to be ascribable to the treatment and not to random or systematic errors [49].

Only three RCTs about the early treatment of open bite were available [23, 28, 40]. However, randomization process was not described.

Sample size was judged as adequate in six studies [21, 27–29, 33, 34]. In the others, it was partly sufficient or insufficient implying low power and high risk to achieve insignificant outcomes.

Previous estimation of sample size was done by two authors [28, 34], but only one [34] described how it was calculated.

The selection description was adequate or fair in all studies except one [21].

The number of dropouts was declared in four studies [17–19, 27], and it was low.

All the trials provided a clear description of the type and duration of the intervention.

The methods used to detect the treatment effects were valid in 12 studies [16, 18, 19, 22, 30–35, 38, 41] and partly valid in ten [17, 20, 23, 24, 27–29, 36, 37, 40].

Some studies [21, 27, 29, 39] lack of an adequate untreated control group probably due to the difficulty in gathering many patients with open bite or the lack of ethical rationale to leave these patients untreated.

Some trials used patients who refused orthodontic therapy [16, 20, 34] or longitudinal data of untreated individuals enrolled in published growth studies [21, 29, 30, 35, 37, 41] as control group.

Weinbach [39] compared the treatment with published cephalometric standards [50].

In one study [21], the method was considered not valid since participants of the groups were not matched according to their dento-skeletal characteristics but just according to age and sex. In another one [39], the appliance was not used exclusively in patients with anterior open bite and there was not a valid control group.

Groups examined by Kiliaridis [17] and Doshi [23] had a too wide age range with subjects treated in the permanent dentition; Pedrin [27] considered too wide ranges of open bite and MPA angle. In two studies [20, 29], treated and control subjects did not have the same age at the beginning and were not observed for the same amount of time which could have influenced cephalometric evaluation of changes.

In six studies, two interventions were tested at the same time, e.g., MBB or RMI and LSE [24], crib and VCC [27, 28], FR and LSE [36, 37, 40], so the results can be attributed either to one or to the other.

SLBB was reported to break frequently in two studies [18, 23]. Doshi did not specify if the treatment was stopped or the appliances were replaced [23]. Kuster and Ingervall replaced the appliances to the patients [18].

Kiliaridis [17] interrupted the treatment earlier than planned and changed the experimental design because of side effects. This did not allow to perform statistical evaluations of the results.

The analysis of the stability of treatment results can not be considered adequate in most studies, in fact follow-up periods were too short [23, 30, 31, 38] and some patients wore contentions while others did not [30, 31], besides some authors applied multibracket appliances during the follow-up [21, 35].

Nineteen studies [17–24, 27–35, 38, 41] included a method error analysis, and only one had blind outcome assessment [17].

Furthermore, five studies declared a power analysis [24, 30, 31, 34, 35].

Fourteen studies used proper statistical methods [18, 20, 22, 29–37, 40, 41]. Among the remaining studies, one did not report any statistics [17], whereas in the others, statistics was inadequate, e.g., parametric tests used in insufficient sample size [16, 19, 23, 24, 39], paired *t* test used improperly to compare changes between groups [21, 27, 28], and inadequate level of significance [38].

## Conclusions

1. Just three RCTs in early treatment of anterior open bite were available.
2. CCTs and CTs indicated the effectiveness of the treatment of anterior open bite in the mixed dentition in improving the overbite.
3. Twenty studies also reported favorable skeletal effects.
4. Studies showed a lack of standardization, important methodological limitations, and shortcomings. The quality level of the studies was not sufficient to draw any evidence-based conclusions. Thus, these results must be viewed with caution.

To determine which treatment is the most effective for early correction of skeletal open bite with a reliable scientific evidence, RCTs with sufficient sample size and more rigorous methodology are required. Future studies should also evaluate stability with a longer follow-up, as well as analysis of tolerability, costs, side effects of the interventions, and patient satisfaction. Diagnostic criteria for anterior open bite should be standardized, and the interventions should be tested to each type of anterior open bite: skeletal or dental. Besides cephalometric measurements, masticatory, swallowing, respiratory functions, maxillary and mandibular growth and measurements, and facial analysis should be evaluated to test the validity of the interventions.
